# Supplementary material for: A neuromorphic processor with on-chip learning for beyond-CMOS device integration
Source: Nat Commun. 2025 Jul 11;16:6424. doi: 10.1038/s41467-025-61576-6 (PMC12254410; doi:10.1038/s41467-025-61576-6)
Supplement: Supplementary file 1 — Supplementary Information [file 41467_2025_61576_MOESM1_ESM.pdf]

A Supplementary Material

A.1 Signal monitoring

TEXEL provides several methods to observe the internal state of various components, circuits and signals. There are three distinct monitoring methods shown in Table 1 under the "Domain" column. The first are analog outputs, they provide access to real time signals measurable by an oscilloscope or Analog Digital Converters (ADCs). The second monitoring method are digital output pins which expose internal digital signals. The third is the asynchronous sADCs interface.

When using the sADC bank, the current under observation is mirrored inside a circuit (described in depth in [1]) that generates a spike rate proportional to the current magnitude. The spikes are propagated through an encoder interfaced with a dedicated 5 bit AER bus. The sADC bank allows the user to monitor 49682 currents, of which 24 simultaneously. Structures that convert currents into spikes for monitoring are popular solutions in literature with several known implementations [1–4]. In many cases, the signal under observation can be selected across synapses and neurons. This is denoted by the "Mux" column in Table 1.

Figure 3 offers an example of how the monitoring methods can be used. In the first row, for example, the presynaptic trace current (I_{PRE}) of a synapse is shown while it receives an input spike train. The synapse has been selected among all the available synapses by setting a register inside the chip through the input AER bus. I_{PRE} is recorded using the sADC and the spiking activity of the sADC is transmitted through the dedicated AER bus. The spikes recorded by the Microcontroller (μC) are used to reconstruct the dynamics of the signal. This is done by finding the Interspike Interval (ISI) of the spike train and taking the reciprocal to calculate the instantaneous spike rate at the corresponding spike time. The resulting current proxy is visible in Figure 3a, using the instantaneous firing rate. The same procedure is repeated for the I_{POST} and the $\mathrm{Ca}^{2+}_{\mathrm{below}}$ current traces, depicted in the same Figure 3a. It is noted that the sADC spiking output is able to capture dynamics on three different time scales effectively ($10\,\mathrm{ms}$ for I_{PRE}, $100\,\mathrm{ms}$ for I_{POST} and $1\,\mathrm{s}$ for the $\mathrm{Ca}^{2+}_{\mathrm{below}}$ trace). In the second row of Figure 3a, we see another example of the monitoring capability of the chip: $\mathrm{V}_{\mathrm{mem}}$, the membrane voltage of the neuron. Using the same procedure explained for the synapse, a specific neuron is chosen for monitoring. This outputs the membrane potential of the neuron on a Bayonet-Neill-Concelman (BNC) cable. For synaptic signals, V_{w} can be observed in the last row of Figure 3a, by selecting it through a monitoring register.

A.2 sADC

On the TEXEL chip 49682 currents can be monitored, of which 24 simultaneously. This is possible thanks to the implementation of the sADC [1]. The circuit follows a mixed signal approach, where the analog block continuously interacts with the asynchronous digital block in a way inspired by mixed-signal implementations of spiking neurons. The working principle of the sADC is as follows: the current under monitoring is mirrored from the circuit and fed in the input of the sADC. This current is directed towards the negative input node of a Operational Amplifier (OPAMP), connected through a capacitor $\mathrm{C}_{\mathrm{mem}}$, to the OPAMP's output, generating a negative feedback loop. The negative feedback loop, under ideal conditions, allows for the creation of a virtual ground: the negative input node of the OPAMP stabilizes its voltage close to $\mathrm{ref\_h}$, regardless of the input current received, while allowing the input current to charge $\mathrm{C}_{\mathrm{mem}}$. Charging the capacitor with said current, while the transistor gate (M_1) stays at a fixed voltage, results in an increasing output voltage, which is sensed by a the subsequent circuit. This circuit is composed of a hysteresis-equipped Operational Transconductance Amplifier (OTA) which implements a threshold function. Here, the input voltage is compared to a fixed bias, and only when the input is above a certain voltage $\mathrm{CF}_{\mathrm{REF-L}} + V_{\mathrm{HYS}}$, the output changes its digital state. The switch of the output state activates the asynchronous digital interface (HS), generating a request for a spike event. Once the circuit receives the acknowledgement signal from the subsequent digital block, the reset of the integrated current begins: a digital pulse completely discharges capacitor $\mathrm{C}_{\mathrm{refr}}$, which is then promptly charged back by a constant current $\mathrm{CF}_{\mathrm{PWLK}}$. The time taken by this capacitor to be charged sets the refractory period of the circuit. During this time, in fact, the capacitor $\mathrm{C}_{\mathrm{mem}}$ has its terminals shorted by an active transistor (M_1), inhibiting the ability to charge with input currents. When no acknowledgement is detected, a pull-up is actively keeping the capacitor $\mathrm{C}_{\mathrm{refr}}$ charged. The behaviour of the sADC versus an input current can be seen in Figure S1b, where, using a programmable DAC, the input current has been swept logarithmically between $1\,\mathrm{pA}$ to $1\,\mathrm{nA}$. One can notice the very wide range of frequency at the output that demonstrates the ability of the circuit to monitor a very wide range of input currents.

The tuning parameters for the circuit are:

- EN: a digital flag determining whether the sADC should receive inputs from the monitored signals or from $\mathrm{off\_bias}$.
- $\mathrm{off\_bias}$, a fixed bias current alternative to the input current.

Name	Description	Type	Domain	Port	Mux
I_{DAC}	Current of a single DAC (for calibration)	Current	Frequency	sADC	-
I_{PRE}	Pre trace of the plastic synapse	Current	Frequency	sADC	SYN
I_{SO}	Second order trace of the Ca^{2+} SoDPI trace	Current	Frequency	sADC	NRN
I_{POST}	Post trace of the neuron	Current	Frequency	sADC	NRN
$I_{\text{P-LEFT}}$	Current of the plastic left synapse	Current	Frequency	sADC	SYN
$I_{\text{P-RIGHT}}$	Current of the plastic right synapse	Current	Frequency	sADC	SYN
$I_{\text{S-EXC}}$	Current of the static excitatory synapse	Current	Frequency	sADC	NRN
I_{AHP}	Adaptive current of the neuron	Current	Frequency	sADC	NRN
I_{FO}	First order trace of the Ca^{2+} SoDPI trace	Current	Frequency	sADC	NRN
$I_{\text{S-INH}}$	Current of the static inhibitory synapse	Current	Frequency	sADC	NRN
V_{MEM}	Membrane voltage of the neuron	Voltage	Analog	BNC	NRN
V_{W}	Analog weight of plastic synapse	Voltage	Analog	BNC	SYN
I_{DAC}	Current of a single DAC (for calibration)	Current	Analog	BNC	-
Ca^{2+}_{ABOVE}	Ca^{2+} above high threshold	Voltage	Digital	Pin	NRN
Ca^{2+}_{BELOW}	Ca^{2+} below low threshold	Voltage	Digital	Pin	NRN
$\text{POST}_{\text{ABOVE}}$	Post trace above high threshold	Voltage	Digital	Pin	NRN
W_{SYN}	Digitized V_{W}	Voltage	Digital	Pin	SYN
	**Device Monitoring**				
$I_{\text{DEV-NEG}}$	Current from negative device	Current	Frequency	sADC	SYN
$I_{\text{DEV-NORM}}$	Current from normalizer circuit	Current	Frequency	sADC	SYN
DEV_{READ}	Device read pulse	Voltage	Digital	Pin	SYN
$\text{DEV}_{\text{WRITE}}$	Device write pulse	Voltage	Digital	Pin	SYN
DEV_{INT}	Device interrupt flag	Voltage	Digital	Pin	SYN
$\text{DEV}_{\text{STATE}}$	Device state	Voltage	Digital	Pin	SYN

Supplementary Table 1: **Signals that can be monitored on the TEXEL chip.** The table is divided into three blocks: current signals observable through the spiking output of the sADCs; voltage and current outputs measurable through BNC connectors; and digital flags measurable on output pins.

- ref_h: The voltage at which the virtual ground should be set. This voltage shifts the low node of the capacitor C_{mem}.
- bias: the current at which the OPAMP should be biased: it defines the strength of the feedback loop (so the ability of the circuit to keep the virtual ground to a specific voltage regardless of the input current magnitude and speed).
- ref_l: The voltage at which the capacitor's positive node is compared in the OTA.
- hys: the current deciding the hysteresis value of the OTA. This defines how much capacitor positive node should be offset with respect to ref_l, to elicit a spike, such that $\text{V}_{\text{mem}} > \text{ref\_l} + \text{V}_{\text{hys}}$.
- pwlk: the leakage of the refractory transistor, this parameter sets how long should the circuit wait before being able to integrate current again.

A.3 DAC

Each core incorporates a fully programmable 94-channel 12-bit DAC, capable of generating reference currents and parameters ranging from $0.5\,\text{pA}$ to $2.2\,\mu\text{A}$, inspired by the design proposed in [5]. These parameters serve to configure the operation settings for the neuron, synapse and learning circuits, as well as control the timing of the device interface.

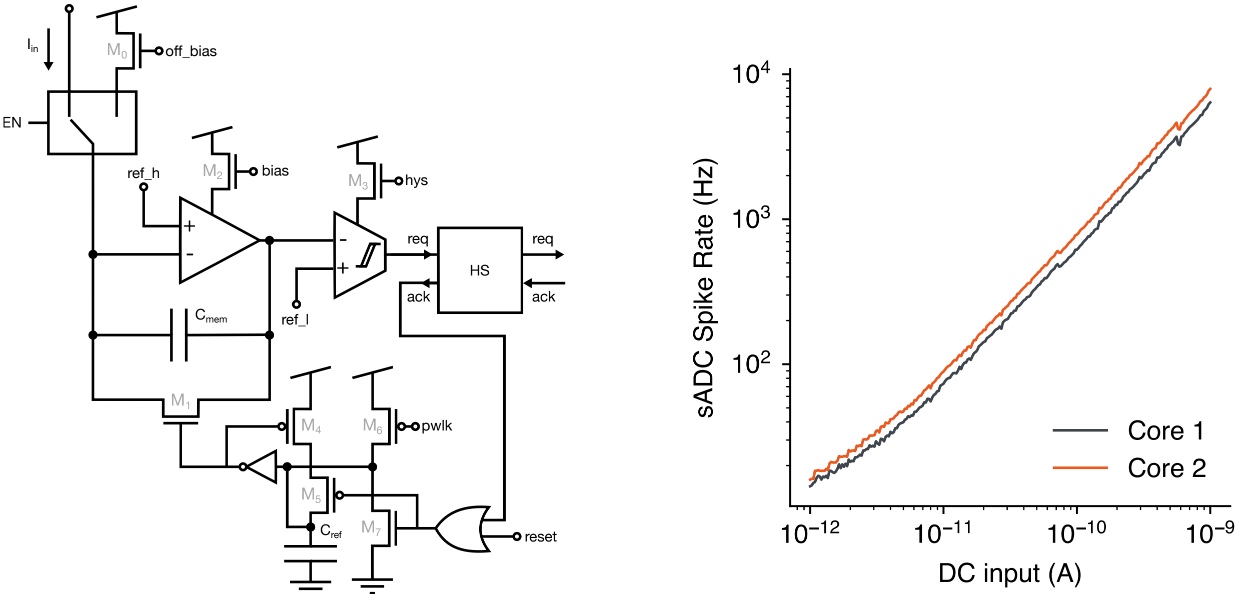

(a) sADC schematic

(b) sADC response

Fig. S1: **sADC circuit and the measurements of the spike rate in response to DC input. a)** sADC schematic with biases labelled. The input current is converted to a spikes and is transmitted via the handshake (HS) block and encoded as an address representing the signal being monitored by the sADC. **b)** Spiking response of two sADC circuits (one on each core) in response to a logarithmic sweep of DC input current from an on-chip DAC. The mapping between current and spike rate obeys a power law, these measurements show how the spike rate of an sADC can be used to infer the magnitude of its input current. The core-to-core deviation is due to mismatch.

The DAC is comprised of three components: first, the configuration storage, which is part of the digital blocks. Second, the reference current generator, responsible for producing six reference currents. Third, the 1T-2T current dividers to generate channel currents from these references. The reference current segment employs a subthreshold CMOS and resistor Proportional To Absolute Temperature (PTAT) source, augmented by a current divider block that incorporates a resistor in the divider to function as a Complementary To Absolute Temperature (CTAT) source. Together with the PTAT, this combination reduces the temperature sensitivity. The resulting current is directed into scaling current mirrors and conveyors to generate scaled base currents (master currents). In this instance, master currents include values of $2.2\,\mu\text{A}$, $0.29\,\mu\text{A}$, $36\,\text{nA}$, $4.5\,\text{nA}$, $0.57\,\text{nA}$, and $70\,\text{pA}$. For each channel, one of these currents is chosen. The resulting current is then passed to a finer division stage of 8 bits, providing 256 levels, with the last selection per channel determining whether the current is sourced by an nFET or a pFET. The fine division stage is composed of MOSFETs substituting resistors in the common 1R-2R DAC circuit (here called 1T-2T). Due to the fact that the current divider employs MOSFETs instead of resistors, the saturation condition of the transistors need to be guarded. The result of the violation of the saturation condition of the MOSFET is the DAC not being entirely monotonic.

A.4 Device operation & integration

A.4.1 Synapse controller

We conducted chip measurements to verify the functionality of the synapse controller. The synapse controller constitutes circuitry at each synapse which manages cases where read and write operations overlap. Three scenarios of read-write interactions are examined through read and write protocols, with digital pins capturing read, write, and interrupt pulses, and weight changes monitored via analog channels. The controller appropriately prioritizes read operations over writes, as evidenced by the detection of interrupt flags when a read coincides with a write pulse, and then executes the write; this ensures correct device operation (Fig. S3).

A.4.2 Continuous read

In addition to the aforementioned device operation mode, the TEXEL platform can be configured to a "continuous read" mode. In this mode the READ signal is permanently set to high such that the drain of the device is held at $V_{\text{read,D}}$, the gate is held at $V_{\text{read,G}}$ and the source is connected to both input branches of the normalizer circuit (Fig. 8). This

READ state is mutually exclusive with respect to memristive device writing signals (POT/DEP). In this mode the IDLE signal becomes obsolete and is held at ground. This “continuous read” mode would be used in the case for which the memristive device capacitance is high and potentially outside “compatibility” range derived from simulations.

A.4.3 Transition-metal oxides

Two-terminal memristive devices consisting of one or more layers of transition metal oxides are widely used for neuromorphic systems, especially for emulating synaptic functions [6]. Here we evaluate three-layer memristive device stacks for their integration into the TEXEL platform. The layer sequence of the memristive device considered for this purpose is HfO_x /Al_2O_3 /TiO_2, embedded between an Au contact layer and the TiN bottom electrode [7]. Here, the HfO_x is responsible for the memristive behavior, the Al_2O_3 changes the interface properties and the TiO_2 layer is advantageous because it forms well-defined interfaces with the TiN electrode and the Al_2O_3 intermediate layer. Furthermore, the Al_2O_3 layer controls the generation of oxygen vacancies and thus serves to limit the current. This is particularly important for integrating the devices into circuits in order to operate the devices without a current compliance.

The stoichiometry of the HfO_x layer is decisive for the resistive switching mechanism [7]. Particularly for sub-stoichiometric oxides (x between 1.5 and 1.8), filamentary switching is observed, while stoichiometric oxide layers ($x = 2$) have an interface-based switching mechanism. However, this leads to different device properties. Gradual resistance switching is observed in interface switching devices, while devices based on filamentary switching exhibit a more abrupt switching characteristic. In the latter, however, multi-level resistance states can be achieved by careful design of the oxygen-vacancy filament. However, the two classes of devices have different requirements that need to be considered when integrating them into the TEXEL platform, which we have analyzed below. Both types of switching devices were fabricated in a thin-film technology using reactive DC magnetron sputtering. This was used to deposit the layers of the device stack with the following thicknesses: HfO_x has a thickness of $3\,\mathrm{nm}$, Al_2O_3 of $2\,\mathrm{nm}$ and TiO_2 of $15\,\mathrm{nm}$. The device electrodes are electrically insulated by a $180\,\mathrm{nm}$ thick SiO_2 layer, encapsulating the functional layers. A $30\,\mathrm{nm}$ thick Au layer defines the top electrode and are used to define the active device area. Further details on the device fabrication can be found in [7].

Interface switching devices: The resistance values for this class of devices are between $0.7\,\mathrm{M\Omega}$ and $290\,\mathrm{M\Omega}$, depending on the concentration of oxygen vacancies in the active memristive HfOx layer. Here, $R_{\mathrm{on}}/R_{\mathrm{off}}$ ratios of up to 10^{-3} are achieved. The switching voltages required for this are $2.5\,\mathrm{V}$ or $3.5\,\mathrm{V}$ for setting the devices and $-1.5\,\mathrm{V}$ or $-3\,\mathrm{V}$ for resetting. This corresponds to current values of $3.6\,\mathrm{\mu A}$ and $10\,\mathrm{nA}$ as well as $-2.1\,\mathrm{\mu A}$ and $-10\,\mathrm{nA}$. In other words, values that are compatible with the TEXEL platform (Fig. 4c). However, these values are dependent on the device area and were determined for an area of $20\,\mathrm{\mu m}^2$. For a direct integration of these devices on the contact areas shown in Fig. 8a, a reduction of the device area by a factor of about 10 is necessary. However, this would be accompanied by a moderate increase in resistance. This can be estimated from a resistance value in the off state of $20\,\mathrm{M\Omega}$ for the current area size to $40\,\mathrm{M\Omega}$ - $50\,\mathrm{M\Omega}$ if the device area size is reduced by a factor of 10. Values that the TEXEL platform allows.

Another important device parameter that must be determined and adapted for the integration of the devices into the TEXEL platform is the device/layer capacitance. The layer capacitance of the transition metal oxides in the layer thickness range used here can be estimated in the range of $10^{-14}\,\mathrm{F\,\mu m^{-2}}$ [8], which fulfils the requirements of the TEXEL platform given in Fig. 4d.

Filamentary switching devices: For filamentary memristive devices, the resistance values of the off resistance are in the range of $0.16\,\mathrm{M\Omega}$ - $80\,\mathrm{M\Omega}$ depending on the concentration of oxygen vacancies in the HfO_x layer [7]. Voltages from -1.5–$-3.0\,\mathrm{V}$ are required for setting the devices, while resetting requires voltages in the range 2.5–$3.0\,\mathrm{V}$. This can be converted into current values in the range $-20\,\mathrm{\mu A}$ to $-20\,\mathrm{nA}$ for the setting process and $15\,\mathrm{\mu A}$ to $40\,\mathrm{nA}$ for the resetting process. The $R_{\mathrm{on}}/R_{\mathrm{off}}$ ratio with is 10^{-1}–10^{-2}, slightly smaller compared to interface switching devices, but fulfils the requirements of the TEXEL platform very well, as shown in Fig. 4c. Capacitance is determined by the device area as well as the filament area. The relevant size for integration is the device area capacitance, which we assume to be $10^{-14}\,\mathrm{F\,\mu m^{-2}}$ as in the case of interface switching devices. The scaling of the device area has no influence on the resistance values. However, an inertial forming step is required for these devices, which requires voltages of up to $\pm 5\,\mathrm{V}$, which is compatible with the TEXEL platform.

A.4.4 Ferroelectric hafnia

Two- and three-terminal synaptic weights based on ferroelectric hafnia are evaluated for their integration on the TEXEL platform. In the two-terminal configuration, the current flows through the ferroelectric layer, which requires the layer thickness to be scaled while maintaining a high polarization. The materials were specifically developed on the XFAB $180\,\mathrm{nm}$ technology, replicating the conditions for integration on the TEXEL platform. The conductivity and the dynamic

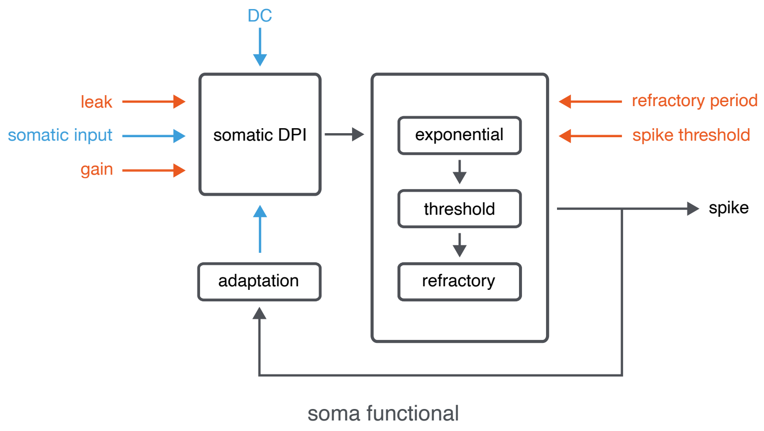

Fig. S2: **The functional architecture of the neuron on the TEXEL chip.** The neuron features a somatic DPI that integrates input from both DC and synaptic sources, with circuits for thresholding, spike generation, refractory period, and positive feedback to mimic biological spiking neurons. An adaptation mechanism can be enabled to modulate spike frequency. Orange inputs represent tunable biases, and blue elements indicate current sources.

range of the BEOL-integrated synaptic weights were found to differ from the same weights nanofabricated on dummy Si wafers [9]. The $2\,\mathrm{V}$ required to operate the synaptic weights fall well in the range available in TEXEL. The dynamic range falls between 1 and 10, for which the optimal resistance is predicted to be in the $10\,\mathrm{G\Omega}$ range. It would result in an average current sourced by the differential normalizer synapse of two thirds of $\mathrm{norm\_bias}$. The scalability of the resistance with the area allows to adapt the design to the current requirement: an ideal resistance of $10\,\mathrm{G\Omega}$ is obtained by scaling the device to $10\,\mathrm{\mu m}^2$.

In the three-terminal configuration (FeFET or thin-film transistor) the ferroelectric gate is integrated prior to the semiconducting oxide channel. The materials optimized for the fabrication of two-terminal devices on TEXEL were evaluated for three-terminal devices, i.e. with an increased gate thickness up to $10\,\mathrm{nm}$. In test circuits, the ferroelectric switching of the capacitors was demonstrated through the same interconnects and transistors that on the TEXEL platform [10]. The saturation for the ferroelectric switching is obtained for $\pm 4\,\mathrm{V}$, in line with the device requirements.

The CMOS-compatibility translates in the absence of degradation of the front-end electronics during the back-end integration of the synaptic weights. For the ferroelectric technology presented above, the critical steps are:

1. The deposition of a functional tungsten oxide layer at $375\,^{\circ}\mathrm{C}$ under an oxidizing plasma.
2. The crystallization of hafnia in the ferroelectric phase.

It uses a flash lamp annealer applying a $20\,\mathrm{ms}$ long energy pulse of $90\,\mathrm{J\,cm^{-2}}$, at a temperature of $375\,^{\circ}\mathrm{C}$. The XFAB $180\,\mathrm{nm}$ MOSFET characteristics prior and after the ferroelectric device integration were compared and did not show significant changes [9]. These preliminary results represent a first milestone towards the evaluation of two- and three-terminal synaptic weights based on ferroelectric hafnia using the TEXEL neuromorphic processor.

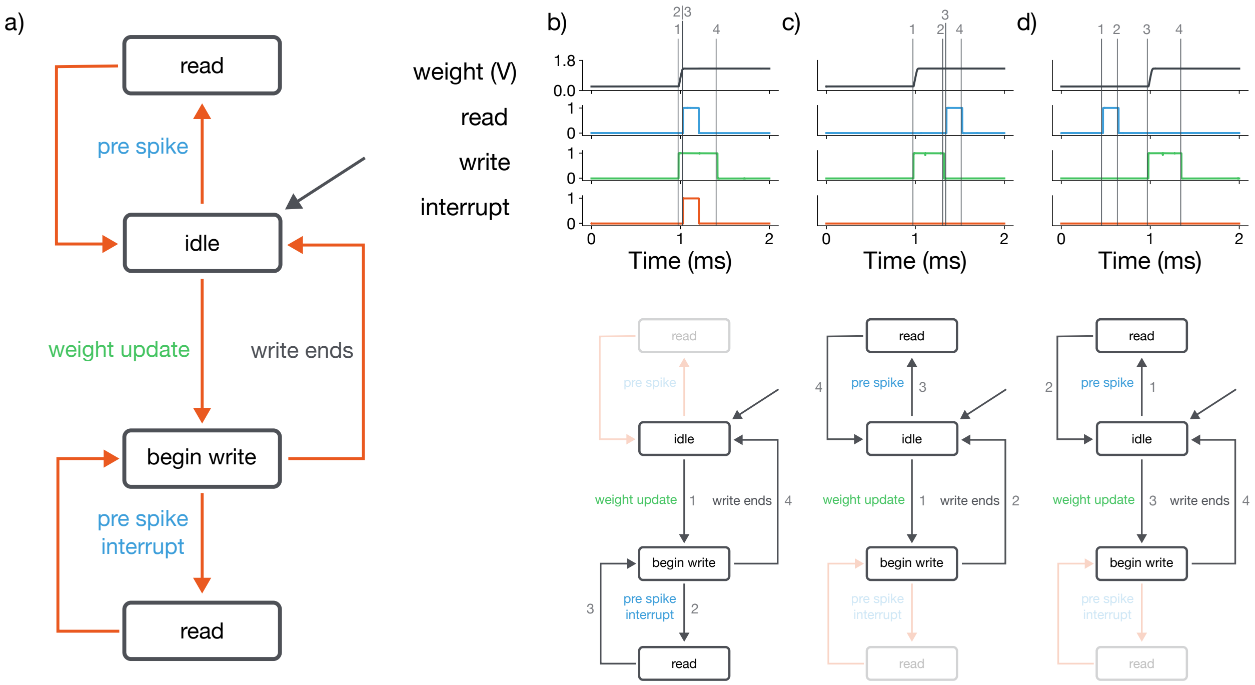

Fig. S3: **Device synapse controller state machine and silicon measurements of the digital flags. a)** A state diagram delineating the internal states of the digital logic governing the addressing protocol for devices on TEXEL. Each row represents a potential combination of presynaptic spikes, postsynaptic spikes, and the state update of synaptic weights. When a presynaptic spike is present, the synaptic weight is read. If a weight update is triggered by either a pre or postsynaptic spike, the logic initiates the writing protocol for the differential device setup. The sequence of these events is unproblematic unless a write and read request are concurrently issued. In such a scenario, a read request takes precedence, and any write request is temporarily halted to allow for the read to take place. Following the reading of the devices, the write process is subsequently executed. **b)** Silicon measurements of digital flags raised by the device controller circuitry located within each synapse. A device read occurs at the same time as a device write, in this case an interrupt flag is raised such that a read can be prioritised and write is subsequently executed. **c)** A write occurs, due to a synaptic weight change, and a read follows. No interrupt flag is raised. **d)** A read, due to a presynaptic spike, occurs before a write. No interrupt flag is raised.

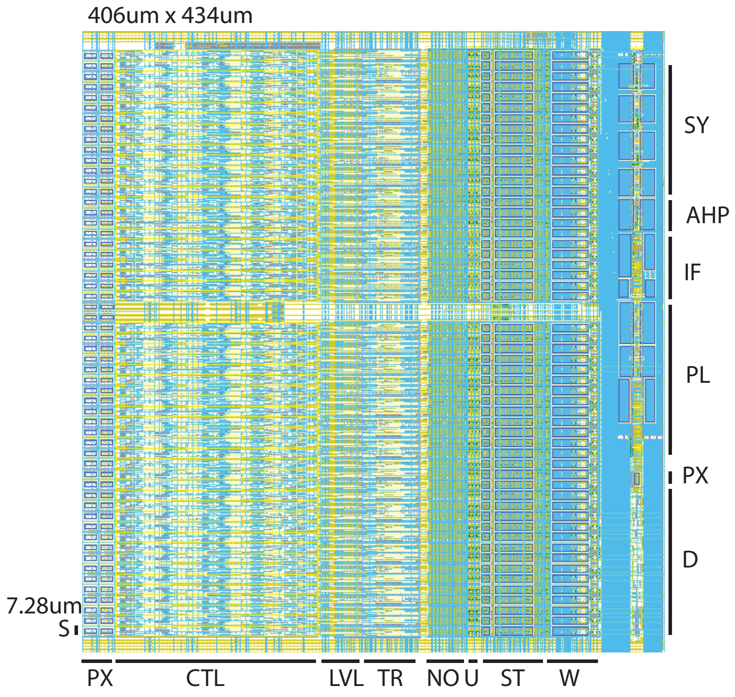

(a) Neuron block footprint

Abbreviation	Name	Area %
	**Synapse**	
S	Synapse	-
PX	Pulse Extender	5.4
CTL	Device Controller	31.5
LVL	1.8-5V Level Shifters	5.8
TR	Device Interface	9.9
NO	Normalizer	6
U	Post Synaptic Current	2.2
ST	Synaptic Trace	10.6
W	Bistable Weight/Update Logic	4
	**Soma**	
SY	PSC Filter	0.4
AHP	Spike Frequency Adaptation	0.34
IF	Leaky Integrate and Fire	
PL	Post Learning Traces	0.43
D	Neuron Digital Logic	0.41
	Wiring and Power	23.02

(b) Key

Fig. S4: **The neuron block macro of TEXEL, detailing the location and footprint area of the circuits. a)** The footprint of the neuron block with associated labels and sizings. **b)** Table defining the abbreviations and providing the % of area of the neuron block macro they occupy.

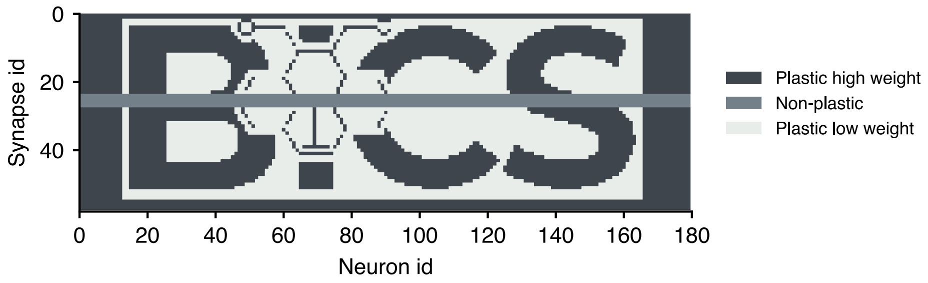

Fig. S5: **Readout of a weights matrix programmed onto the plastic synapses of TEXEL.** Each of the 54 plastic synapses has the capability to exist in either a high or low state, effectively storing binary information. A weights matrix can be programmed onto the chip, making it suitable for inference tasks, device operation, and testing; independent of on-chip learning.

Supplementary References

[1] E. Voulgari, M. Noy, F. Anghinolfi, F. Krummenacher, and M. Kayal, "Sub-picoampere, 7-decade current to frequency converter for current sensing", in *2015 IEEE 13th International New Circuits and Systems Conference (NEWCAS)*, 2015, pp. 1–4. DOI: 10.1109/NEWCAS.2015.7182071.

[2] F. Corradi and G. Indiveri, "A neuromorphic event-based neural recording system for smart brain-machine-interfaces", *Biomedical Circuits and Systems, IEEE Transactions on*, vol. 9, no. 5, pp. 699–709, 2015. DOI: 10.1109/TBCAS.2015.2479256.

[3] N. Qiao and G. Indiveri, "An auto-scaling wide dynamic range current to frequency converter for real-time monitoring of signals in neuromorphic systems", in *2016 IEEE Biomedical Circuits and Systems Conference (BioCAS)*, 2016, pp. 160–163. DOI: 10.1109/BioCAS.2016.7833756.

[4] B. V. Benjamin, R. L. Smith, and K. A. Boahen, "A low thermal sensitivity subthreshold-current to pulse-frequency converter for neuromorphic chips", *IEEE Journal on Emerging and Selected Topics in Circuits and Systems*, vol. 13, no. 4, pp. 956–964, 2023. DOI: 10.1109/JETCAS.2023.3321105.

[5] T. Delbrück and A. V. Schaik, "Bias current generators with wide dynamic range", *Analog Integrated Circuits and Signal Processing*, vol. 43, no. 3, pp. 247–268, 2005. DOI: 10.1007/s10470-005-1606-1.

[6] M. Ziegler, C. Wenger, E. Chicca, and H. Kohlstedt, "Tutorial: Concepts for closely mimicking biological learning with memristive devices: Principles to emulate cellular forms of learning", *Journal of Applied Physics*, vol. 124, no. 15, p. 152 003, 2018. DOI: 10.1063/1.5042040.

[7] S. Park, B. Spetzler, T. Ivan, and M. Ziegler, "Multilayer redox-based HfOx/Al2O3/TiO2 memristive structures for neuromorphic computing", *Scientific Reports*, vol. 12, p. 18 266, 2022. DOI: 10.1038/s41598-022-22907-5.

[8] M. Hansen, M. Ziegler, L. Kolberg, R. Soni, S. Dirkmann, T. Mussenbrock, and H. Kohlstedt, "A double barrier memristive device", *Scientific Reports*, vol. 5, p. 13 753, 2015. DOI: 10.1038/srep13753.

[9] L. Bégon-Lours, S. Slesazeck, D. F. Falcone, V. Havel, R. Hamming-Green, M. M. Fernandez, E. Morabito, T. Mikolajick, and B. J. Offrein, "Back-end-of-line integration of synaptic weights using HfO2/ZrO2 nanolaminates", *Advanced Electronic Materials*, p. 2 300 649, 2024. DOI: 10.1002/aelm.202300649.

[10] R. Hamming-Green, M. S. Ram, D. F. Falcone, B. Noheda, B. J. Offrein, and L. Bégon-Lours, "Multi-level, low-voltage programming of ferroelectric HfO2/ZrO2 nanolaminates integrated in the back-end-of-line", in *2024 8th IEEE Electron Devices Technology & Manufacturing Conference (EDTM)*, IEEE, 2024, pp. 1–3. DOI: 10.1109/EDTM58488.2024.10511719.
